# Extensive Superior Vena Caval Territory Thrombosis and Pulmonary Embolism: A Rare Clinical Entity of Haemorrhagic Pancreatitis

**DOI:** 10.1155/2020/8072307

**Published:** 2020-05-24

**Authors:** P. Thineskaran, K. Abiramy, T. Kumanan, V. Sujanitha, N. Suganthan, G. Selvaratnam

**Affiliations:** University Medical Unit, Teaching Hospital, Jaffna, Sri Lanka

## Abstract

Acute haemorrhagic pancreatitis is a severe form of pancreatitis often encountered in ethanol abuse. Extensive venous thrombosis resulting in pulmonary embolism is a rare presenting clinical entity of acute haemorrhagic pancreatitis. Here, we report a young male with an extensive deep vein thrombosis involving superior vena caval territory associated with haemorrhagic pancreatitis presented with pulmonary embolism managed supportively. Prompt recognition and appropriate intervention of this rare complication would improve the outcome in patients with acute pancreatitis.

## 1. Introduction

Acute pancreatitis is defined as “an acute inflammation of the pancreas, characterized by local inflammation that can trigger a systemic inflammatory response and it mostly occurs due to alcohol.” Extensive deep venous thrombosis and pulmonary embolism are rare but life-threatening complications of acute pancreatitis. Hypercoagulable state in acute pancreatitis is due to release of pancreatic proteolytic enzymes that cause proteolytic damage and inflammation of the vessels. Here we report a case of severe haemorrhagic pancreatitis complicated with extensive deep vein thrombosis and pulmonary embolism in a young male.

## 2. Case Presentation

A 25-year-old previously healthy Sri Lankan male presented to the emergency department with a history of painful swelling of the left side on the neck and odynophagia of four days duration. He denied a history of fever and had no symptoms suggestive of upper airway obstruction. He had no significant past medical history to note, except he experienced an episode of severe epigastric pain which was treated as gastritis a year ago. He was an ethanol user, consuming 15 units per week. On examination, he was neither pale nor icteric. His pulse rate was 140 bpm, and blood pressure was 120/70 mmHg. He had tachypnea at rest with a respiratory rate of 32 per minute. Abdominal examination revealed mild tenderness over epigastric region though he denied any history of abdominal pain on admission. A swollen tender area, not moving with swallowing was noted in the lateral aspect of the neck without any palpable cervical lymphadenopathy. Examination of the thyroid gland and oral cavity showed no abnormality except poor oral hygiene. Rest of the systemic examination was unremarkable.

The initial full blood count showed white cell count of 12,580/mm^3^ with predominant neutrophils, haemoglobin of 13.5 g/dL with a haematocrit of 44, and platelets of 206,000/mm^3^. Initial inflammatory markers ESR and C-reactive protein (CRP) were 97 mm/1^st^ hour and 26 mg/L, respectively. Serum amylase was 2180 IU/L, and corrected calcium was 2.21 mmol/L. Random blood glucose on admission was 122 mg/dL, and an ABG showed hypoxemia. Ultrasound scan with Doppler study of his neck showed extensive deep vein thrombosis involving internal jugular veins, subclavian veins, axillary veins, basilic veins, and upper part of superior vena cava. Ultrasound scan of abdomen revealed mixed echogenic area seen below the spleen suspicious for haematoma or haemorrhage into the cyst. At this point, he was diagnosed with an acute pancreatitis complicated with extensive deep vein thrombosis, and he was initiated with enoxaparin along with warfarin targeting an INR of 2.5 in addition to other supportive measures for acute pancreatitis.

**Figure 1:**
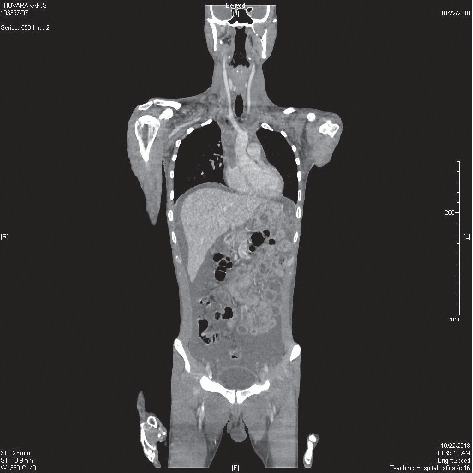
The contrast-enhanced computed tomography of neck, chest, abdomen, and pelvis showed extensive venous thrombosis involving in internal jugular veins, subclavian veins, brachiocephalic vein, and superior vena cava up to the right atrium.

**Figure 2:**
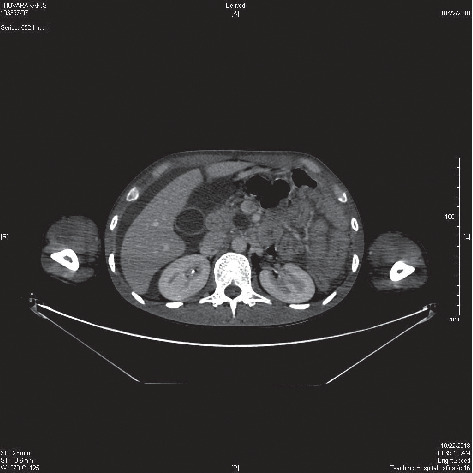
The contrast-enhanced computed tomography of abdomen shows two cystic collections suggestive of pseudo cysts in pancreas.

Subsequent contrast enhanced-computed tomography of chest, abdomen, and pelvis with venogram showed extensive venous thrombosis involving in both internal jugular veins, subclavian veins, bracheocephalic vein, and superior vena cava up to the right atrium (Figure 1) and pulmonary arterial thromboembolism. It also revealed moderate right-sided pleural effusion and gross ascites and features of acute-on-chronic pancreatitis with two cystic collections suggestive of pseudo cyst formation (Figure 2).

The 2D echocardiography showed normal cardiac function with an ejection fraction of 55%. Extensive investigations done on him to identify an alternative cause for his deep vein thrombosis failed to show any positive results. Anticardiolipin antibody, protein C, S, Factor V Leiden mutation, hams test, and urine for haemosiderin were negative. Markers of autoimmune pancreatitis and pancreatic malignancy were normal.

On day 10 of admission, he developed a bout of severe abdominal pain associated with haemodynamic instability. An urgent ultrasonography of abdomen showed bleeding into the cyst, and it was managed with therapeutic aspiration of 2000 ml of haemorrhagic fluid and transfusion of packed cells while withholding the anticoagulant. Analysis of aspirate showed an exudative fluid with an amylase level of 10435 U/L and LDH of 6060 U/L, suggestive of haemorrhagic pancreatitis. Cytological analysis did not reveal evidence of a malignancy. Parenteral antibiotics and other supportive therapies were continued. Anticoagulant was reinstituted after few days. He made a gradual recovery and discharged from hospital on day 24.

On review at a month later, he showed significant clinical improvement with an evidence of resolving deep vein thrombosis.

## 3. Discussion

Acute pancreatitis is an acute inflammatory process of the pancreas that presents with severe abdominal pain with elevated levels of pancreatic enzymes in the blood. Serum amylase and lipase are the important sensitive biochemical markers used to diagnose and monitor acute pancreatitis in clinical practice. The contrast-enhanced computed tomography of the pancreatic system is the widely used preferred diagnostic imaging and used in prognostic assessment of pancreatitis as well.

According to the revised Atlanta classification system, this patient had moderately severe acute pancreatitis. His Balthazar grade was D with a moderate severity. His Acute Physiology and Chronic Health Evaluation II score was 8 points with 8.7% estimated nonoperative mortality. An inherited or acquired thrombophilic state can leads to venous thrombosis. However, this patient had negative test results for any inherited or acquired prothrombotic conditions. Therefore, in this context, it is assumed that his extensive thrombosis involved in deep veins of neck and pulmonary regions were secondary to severe recurrent acute pancreatitis.

The vascular complications are the major cause of morbidity and mortality of acute pancreatitis that are related to haemorrhage resulting from arterial erosion or pseudoaneurysms, ischaemic complications, and venous or arterial complications—specifically splanchnic thrombosis and associated varices [1]. Extensive deep vein thrombosis involving the pulmonary, neck, and calf veins are a rare complication of acute pancreatitis that has been described in few case reports in the literature to date [2]. A case has been reported with acute pancreatitis with ischaemic cerebrovascular accident and pulmonary embolism which was successfully managed with anticoagulation [3]. Hypercoagulable state said to occur due to release of proteolytic enzyme in pancreatic juice in a cyst communicating with the pancreatic duct penetrates into the vascular system that causes proteolytic damage and inflammation of the vessels [4]. The pancreatic elastase is a major role in the development of pulmonary vascular injury after acute pancreatitis. The combination of hepatic dysfunction and hypertrypsinemia (resulting in raised fibrinogen and factor VIII concentrations) and cachexia, immobility also cause hypercoaguable state of acute pancreatitis. Furthermore, the sub sensitivity of contractile response to phenylephrine in both mesenteric and pulmonary rings might be due to the complications of this pathological condition in the early stage of pancreatitis [5].

Prompt investigations and clinical vigilance should be emphasized among clinically suspected acute pancreatitis because accurate diagnosis and timely radiological intervention procedures could reduce mortality. Early institution of intravenous heparin or thrombolysis is recommended to prevent further complications of acute pancreatitis once a diagnosis was confirmed.

## 4. Conclusion

Extensive venous thrombosis is a rare presentation of acute haemorrhagic pancreatitis. Anticipation of this rare complication would aid in early diagnosis, specific therapy, and to prevent fatal pulmonary embolism, a rare but catastrophic phenomenon of thrombosis in patients with acute haemorrhagic pancreatitis.
